# Factors associated with glucose tolerance, pre-diabetes, and type 2 diabetes in a rural community of south India: a cross-sectional study

**DOI:** 10.1186/s13098-016-0135-7

**Published:** 2016-03-08

**Authors:** Matthew Little, Sally Humphries, Kirit Patel, Warren Dodd, Cate Dewey

**Affiliations:** Department of Population Medicine, University of Guelph, Guelph, ON Canada; Department of Sociology and Anthropology, University of Guelph, Guelph, ON Canada; Department of International Development Studies, Menno Simons College, University of Winnipeg, Winnipeg, MB Canada

**Keywords:** Type 2 diabetes, Rural, India, Cross-sectional, Body mass index, Waist to hip ratio, Impaired fasting glucose, Impaired glucose tolerance, Diet, Tobacco

## Abstract

**Background:**

India’s national rural prevalence of type 2 diabetes has quadrupled in the past 25 years. Despite the growing rural burden, few studies have examined putative risk factors and their relationship with glucose intolerance and diabetes in rural areas. We undertook a cross-sectional study to determine the prevalence of impaired fasting glucose (IFG), impaired glucose tolerance (IGT), and type 2 diabetes in a rural area of south India. In addition, we determined which factors were associated with type 2 diabetes.

**Methods:**

We sampled 2 % of the adult population from 17 villages using a randomized household-level sampling technique. Each participant undertook a questionnaire that included basic descriptive information and an assessment of socioeconomic status, physical activity, and dietary intake. Height, weight, waist and hip circumference, and blood pressure measurements were taken. An oral glucose tolerance test was used to determine diabetes status. We used stepwise logistic model building techniques to determine associations between several putative factors and type 2 diabetes.

**Results:**

753 participants were included in the study. The age- and sex-standardized prevalence of IFG was 3.9 %, IGT was 5.6 %, and type 2 diabetes was 10.8 %. Factors associated with type 2 diabetes after adjusting for confounders included physical activity [OR 0.81], rurality [OR 0.76], polyunsaturated fat intake [OR 0.94], body mass index [OR 1.85], waist to hip ratio [OR 1.62], and tobacco consumption [OR 2.82].

**Conclusion:**

Our study contributes to the growing body of research suggesting that diabetes is a significant concern in rural south India. Associated risk factors should be considered as potential targets for reducing health burdens in India.

## Background

Type 2 diabetes mellitus (diabetes) is an international pandemic with high morbidity and mortality and a large economic impact. An alarming 80 % of the global burden lies in low- and middle-income countries (LMICs) [1]. While in urban areas of LMICs, diabetes is recognized as a public health priority and is often well studied; recent prevalence data suggest that diabetes is an increasing problem among rural populations as well [2]. In India, while regional differences exist, national rural prevalence has quadrupled in the past 25 years [3]. A recent large-scale study in a rural community in south India found a diabetes prevalence rate of 7.8 %. The same study found prevalence of pre-diabetes (impaired fasting glucose and/or impaired glucose tolerance) was 7.1 %, representing a population at heightened risk of conversion to overt diabetes [4]. Despite evidence suggesting the growing rural diabetes burden, few studies have examined putative risk factors and their associations with glucose intolerance and diabetes in rural areas. ‘Modernization’ and ‘globalization’ are often blamed, but precise pathways of causation are unclear and understudied [2]. Moreover, rising national prevalence of diabetes is often attributed to urbanization, which results in a disproportionate focus on urban areas and ignores the epidemiological environment of rural regions [3]. Given the inherent diversity of livelihoods, ethnicities, cultures, food habits, and lifestyles within India, there exists a need for research on diabetes and risk factors from different regions across the entire urban/rural continuum to broaden our understanding of the epidemic [5].

We report here the findings of a cross-sectional study in a rural population of northern Tamil Nadu. The study had two principal objectives: first, to establish prevalence rates of impaired fasting glucose (IFG), impaired glucose tolerance (IGT), and diabetes in the research site; and second, to assess associations between diabetes risk and a number of anthropometric, socioeconomic, lifestyle, and dietary factors through multivariate linear and logistic regressions. Such analyses identify potential risk factors that can be further analyzed in case–control and cohort studies. Identifying risk factors of diabetes is crucial for resource prioritization in public health efforts, especially considering the shortage of personnel and resources in rural areas.

## Methods

### Ethics, consent, and permissions

We obtained clearance for the study from a Canadian university ethics board. Permission for the study was granted by the High Commission of India in Ottawa, Canada. Upon arrival to the research site, and prior to the recruitment process, we approached local authorities (*panchayat* councils, local police officials, and hospital medical staff) and sought and obtained permission to carry out the study. Informed verbal consent was obtained from all research participants prior to enrollment.

### Study design and sample

The sampling frame consisted of the entire adult population (>19 years old) of two rural panchayat wards (Anchetty panchayat and Madakkal panchayat), in the Krishnagiri District of Tamil Nadu. The region is comprised of several small villages surrounding the central market village of Anchetty.

All villages within the sampling area were included in the study. We mapped each village, numbered the houses consecutively, and then randomly selected 8 % of households from which to sample one adult individual. Our target was to sample 800 participants, based on a sample size calculation with an estimated diabetes prevalence of 8 %, a precision of 0.02, and a confidence of 95 %. Upon approaching a household, we employed World Health Organization’s (WHO) Kish method to recruit an individual for the study [6]. If an individual declined to participate, we removed them from the household list and employed the Kish method again. If all adults in the household refused to participate, we approached a neighbouring household and repeated the process. Upon receiving consent, we collected basic census details, including age, sex, education, and occupation.

Socioeconomic status (SES) was assessed using a wealth index comprised of a subset of 13 of 29 questions taken from the Standard of Living Index used by the second round of the National Health and Family Survey (NFHS-2) [7]. We selected those questions believed to be most relevant for our study population, including both household and village characteristics. Each attribute was weighted to give a maximum score of 36. Weights of items were developed by the International Institute of Population Sciences in India based on a priori knowledge about the relative significance of the items in determining SES [8]. Due to the rural subsistence nature of the local economy, this asset-based score is considered a more appropriate indicator of SES than education, income, or occupation [8].

We assessed dietary patterns using a validated interviewer-administered semi-quantitative food frequency questionnaire (FFQ) [9]. Individuals were asked to estimate the frequency of consumption (number of times per day/week/month/year) and usual serving size of dishes and food items in the FFQ. A ‘food atlas’ served as a visual prompt for foods and serving sizes. FFQ data was analyzed using EpiNu, a software program that combined frequency of food consumption with corresponding nutrition information to provide detailed data on caloric consumption and average daily macro- and micronutrient intake [10]. Detailed descriptions of this FFQ and data on reproducibility and validity, including a comparison of FFQ estimates to 24-h dietary recalls, have been published elsewhere [9]. All dietary variables except energy intake (kcal/day) were scaled to g/ 1000 kcal to account for differences in energy intake between participants.

Physical activity was assessed using the WHO Global Physical Activity Questionnaire (GPAQ) [11]. The GPAQ evaluates individuals’ physical activity behaviour in four domains: work, travel, and recreation, and sedentary time. The GPAQ was paired with locally relevant photographs depicting ‘moderate’- and ‘vigorous’-intensity work and recreation activities. Physical activity scores were calculated using WHO’s GPAQ Analysis Guide, which provided a total measure of Metabolic Equivalent of Task (MET min/week) [11]. We scaled values to 1 h/day of moderate physical activity for ease of interpretation. Sedentary time was calculated as h/day spent relaxing, watching television, and performing sedentary work duties (e.g. desk work). Television time was assessed as h/day spent watching television.

All previous epidemiology studies in India known to the authors define households as either rural or urban based on a dichotomous typology, which fails to adequately capture the variability inherent in the urban/rural continuum [12]. In order to better capture rurality, we created a rurality index based on Weinert and Boik’s (1995) and adapted for use in India [13]. The index was generated by summing the standardized values of (1) distance to primary health center (in kilometers, assigned half weight), and (2) number of households in home village (assigned full weight). Values were standardized to a mean of zero and a standard deviation of one. The rurality index was therefore a unitless quantitative value assigned to each individual, with a higher value indicating a greater degree of isolation and/or a lower population density of household location.

Height, weight, waist circumference, and hip circumference measurements were taken by a trained nurse using standardized techniques [14]. We calculated body mass index (BMI) and waist-to-hip circumference ratios (WHR). Blood pressure was recorded using a portable OMRON BP-760 electronic blood pressure machine (Omron Healthcare, Hoofddorp, Netherlands). We took two measurements within 5 min on the right arm in the sitting posture, and the mean of the two measurements was recorded.

Glucose tolerance and diabetes status was determined using an oral glucose tolerance test [15]. After an 8-h minimum overnight fast, we measured fasting capillary blood glucose (CBG) using a One Touch Ultra glucometer (Johnson and Johnson, Milpitas, CA, USA). Oral glucose (82.5 g glucose dissolved in 250 ml water, equivalent to 75 g anhydrous glucose) was administered and consumed within 5 min. A 2-h post-load CBG was then collected [14]. CBG was adopted instead of venous plasma glucose estimations due to unavailability of quality-controlled laboratories and difficulties associated with transporting refrigerated blood samples to a central laboratory. Studies have also found higher nonresponse rates associated with venous blood draws [16, 17]. Priya et al. (2011) compared CBG to venous plasma glucose estimation and determined the Pearson’s correlation coefficient was 0.681 (*p* < 0.001) in the fasting state and 0.897 (*p* < 0.001) 2-h post-load [16]. Thus, CBG was viewed as an accurate and acceptable alternative to venous plasma glucose estimation for diabetes screening.

### Definitions

High blood pressure was defined as an individual with systolic blood pressure 140 mmHg or more and diastolic blood pressure 90 mmHg or more [18]. Diabetes was defined as individuals diagnosed by a physician who could provide proof of diagnosis and/or those who had a fasting CBG ≥7 mmol/L (≥126 mg/dl) and/or a 2-h post-load CBG value ≥12.2 mmol/L (≥220 mg/dL) [15, 19]. Impaired fasting glucose (IFG) was defined as a fasting CBG ≥6.1 mmol/L (≥110 mg/dL) and <7 mmol/L (<126 mg/dL) and a 2-h post-load value <8.9 mmol/L (<160 mg/dL) [15]. Impaired glucose tolerance (IGT) was defined as a fasting CBG <7 mmol/L and a 2-h post -load CBG ≥8.9 mmol/L (≥160 mg/dL) but <12.2 mmol/L (220 mg/dL) [15]. Pre-diabetes was defined as having either or both IFG and IGT [19]. Underweight was defined as BMI <18.5 kg/m^2^, overweight was defined as BMI ≥23.0 kg/m^2^ but <25 kg/m^2^, obesity class I was defined as BMI ≥25 and <30 kg/m^2^, and obesity class II was defined as BMI ≥30 kg/m^2^ [20]. Smoking status was categorized as ‘nonsmoker’ or ‘current smoker’. Tobacco consumers were defined as either or both current smokers or current consumers of *paan* (smokeless tobacco).

### Statistical analysis

We identified outliers, cleaned the data, and completed summary statistics in Microsoft Excel 2010. All other statistical analyses were performed in STATA version 13.0 [21]. Prevalence rates were age- and sex-standardized using state-level age and sex data from the 2011 national census [22]. In separate models, univariate interactions between sex and glucose tolerance were analyzed. There were no significant associations between sex and outcome variables, and we therefore present the results for men and women combined. We calculated mean values of descriptive characteristics, socioeconomic position, and dietary intake across categories of glucose tolerance, including normal, pre-diabetes, newly-diagnosed diabetes, and pre-diagnosed diabetes. Values were expressed as the mean ± standard deviation or percentages. One-way analysis of variance (for continuous variables) and the *χ*^*2*^ test (for binary variables) were used to test differences across outcome groups.

We employed a backwards elimination process to build two linear regression models assessing the associations between putative risk factors and 2-h post-load CBG and fasting CBG. All factors were first analyzed in univariate linear regressions (see Appendix 1 for full list of factors). Variables with significant associations (*p-*value <0.2) in univariate analyses were included in an initial multivariate linear regression model. We then methodically eliminated non-significant variables (*p-*value <0.05) from the multivariate model, assuming a lack of confounding if coefficients of all remaining variables did not change by more than 20 % after removal of the potential confounder. Quadratic terms and interaction terms were assessed if there were biological or practical reason to believe they may be significant. BMI and WHR datasets were standardized to a mean of zero and standard deviation of one for ease of comparability. Where linear regressions violated the assumptions of linear models, including normality of residuals and homoscedasticity, various outcome transformations were tested using the Box Cox function in STATA. Following this, models were adjusted if necessary [23]. If no transformations improved the assumptions of the model, we used robust standard errors when assessing the significance of each predictor, as suggested by Pires and Rodrigues (2007) [24].

In a second step, a multivariate multinomial logistic regression model was fitted to examine associations between putative risk factors and pre-diabetes and newly-diagnosed diabetes (i.e. those individuals diagnosed by our study). Normoglycemic individuals were the referent group. Individuals with pre-diagnosed diabetes were excluded to reduce potential reverse causation (i.e. behavioural changes resulting from diagnosis). We employed a backwards elimination model-building process that closely mirrored the methods described above. All putative risk factors were analyzed first in univariate logistic analyses and included in the initial multivariate model if an association existed (*p-*value <0.2). Variables were eliminated if, in the final model, they were not associated with either pre-diabetes or diabetes (*p-*value <0.05). Confounders were identified if they altered remaining coefficients by greater than 20 % after their removal from the model. If a variable was identified as a confounder, it was forced into the final model.

## Results

Of the 812 individuals recruited for the study, 753 participated (341 men and 412 women), of whom 752 (92.6 %) completed a FFQ and 749 (92.2 %) consented to blood sampling. Response rate was 87.4 % among men and 99.2 % among women (*p* < 0.05). Disparity in the response rate was primarily due to migration among local men and thus unavailability at the time of sampling. The mean age was 47 ± 14.7 and the literacy rate was 35.1 %.

The unadjusted prevalence of diabetes was 11.7 %, of which 56.4 % were previously undiagnosed. The overall age- and sex-standardized prevalence of diabetes was 10.8 %. Age- and sex-standardized prevalence of IFG was 3.9 % and IGT was 5.6 %, and overall standardized prevalence of pre-diabetes was 9.5 %. None of the individuals with pre-diabetes were previously diagnosed as such.

Baseline characteristics for the study population by diagnostic category are displayed in Table 1. A significant difference was seen across categories in several descriptive characteristics and wealth attributes. However, the average wealth index and most dietary variables were not significantly different between diagnostic categories. Energy intake was significantly lower for individuals with diagnosed diabetes, perhaps indicating lower energy requirements (due to lower rates of physical activity), or post-diagnosis changes in diet due to doctor recommendations.

**Table 1:** Baseline characteristics of a sample of individuals in rural south India by diagnostic category following an oral glucose tolerance test

Characteristic	Normoglycemia (*n* = 591)	Pre-diabetes (*n* = 64)	Newly-diagnosed type 2 diabetes (*n* = 53)	Diagnosed type 2 diabetes (*n* = 41)	*p-*value for trend
*Descriptive characteristics*					
Mean age	46.2 ± 14.8	48.7 ± 14.1	50.5 ± 13.8	54.1 ± 12.1	0.001
Women (%)	55.0	59.4	47.1	53.7	0.61
BMI (kg/m^2^)	21.2 ± 4.0	23.1 ± 3.9	24.5 ± 4.6	25.3 ± 4.6	<0.001
Waist-to-hip ratio	0.87 ± 0.077	0.90 ± 0.075	0.93 ± 0.10	0.92 ± 0.06	<0.001
Hypertension (%)	25.6	36.5	49.1	57.5	<0.001
Family history of diabetes (%)	6.3	15.6	11.3	56.1	<0.001
Rurality index	0.14 ± 0.94	−0.55 ± 1.06	−0.45 ± 1.07	−0.63 ± 1.02	<0.001
Current tobacco consumer (%)	38.7	42.6	56.6	14.6	0.037
Muslim religion (%)	2.5	4.7	9.4	12.2	0.001
*Wealth attributes*					
Wealth index	10.8 ± 4.5	11.9 ± 5.19	10.6 ± 5.2	11.6 ± 5.3	0.20
Pucca housing (%)	11.7	21.9	15.1	24.4	0.017
In-house tap water (%)	6.6	9.4	15.1	19.5	0.003
*Physical activity habits*					
Physical Activity (h/day of moderate physical activity)	4.37 ± 3.66	3.15 ± 3.55	1.88 ± 2.68	2.63 ± 3.62	<0.001
Sedentary time (h/day)	4.26 ± 2.63	4.98 ± 3.17	5.67 ± 2.98	4.92 ± 2.51	<0.001
Television time (h/day)	1.43 ± 1.26	1.80 ± 1.63	1.52 ± 1.44	1.17 ± 0.95	0.012
Labour occupation (%)	62.6	51.6	55.7	40.5	0.015
Livestock ownership (%)	45.1	29.7	26.4	34.1	0.002
*Dietary intake*					
Current alcohol consumer (%)	48.2	46.8	52.8	26.8	0.051
Total energy intake (kcal/day)	2423.7 ± 758	2241.5 ± 671	2313.3 ± 618	2124.3 ± 504	0.003
Carbohydrates (g/1000 kcal)	180.1 ± 14.1	179.8 ± 14.0	176.7 ± 16.5	175.8 ± 17.1	0.14
Protein (g/1000 kcal)	25.6 ± 1.9	25.5 ± 1.9	25.7 ± 1.9	26.2 ± 1.7	0.25
Total fat (g/1000 kcal)	19.5 ± 5.4	19.5 ± 4.7	20.4 ± 5.2	20.7 ± 5.2	0.39
Dietary fibre (g/1000 kcal)	31.7 ± 11.9	32.6 ± 11.2	30.5 ± 11.2	31.5 ± 12.3	0.82
Dairy products (g/1000 kcal)	79.7 ± 69.7	71.1 ± 51.8	98.8 ± 68.4	89.1 ± 61.1	0.12
Pulses and legumes (g/1000 kcal)	27.3 ± 11.8	29.1 ± 10.7	26.7 ± 12.2	29.6 ± 10.7	0.41
Meat and poultry (g/100 kcal)^a^	3.16 ± 3.9	3.17 ± 3.8	3.34 ± 4.6	2.67 ± 3.22	0.86
Fruits and vegetables (g/1000 kcal)	76.2 ± 48.6	86.0 ± 57.2	73.3 ± 42.6	70 ± 32.2	0.23
Refined grains (g/1000 kcal)	159.5 ± 82.3	147.1 ± 76.9	136.7 ± 79.4	142.4 ± 72.6	0.14

^a^ Meat and poultry intake was low due to the high rate of vegetarianism among the research population and the high relative cost of meat products in comparison to grain products

Table 2 displays the results of the multivariate linear regression model with 2-h post-load CBG as the dependent variable. BMI and WHR were positively associated with 2-h CBG. Physical activity, rurality index, and intake of polyunsaturated fatty acids were negatively associated with 2-h CBG. The untransformed model was heteroscedastic using a Cook-Weisberg test and lacked normality of standardized residuals using a Shapiro–Wilk statistic. We used a negative inverse transformation on the outcome variable, which solved the heteroscedasticity problem but residuals still lacked normality. We therefore used robust standard errors when assessing the significance of each predictor [24]. The adjusted R-squared value for the final model was 0.18.

**Table 2:** Factors associated with 2-h post-glucose-load capillary blood glucose during an oral glucose tolerance test in a sample of individuals in rural south India

Variable	Coefficient	Robust standard error	*p-*value
Constant	−0.23	0.021	<0.001
Physical activity (h/day moderate activity)	−2.7 × 10^−3^	04.8 × 10^−4^	<0.001
Body mass index (standardized to one unit SD)	5.1 × 10^−3^	2.2 × 10^−3^	0.022
Waist-to-hip ratio (standardized to one unit SD)	5.7 × 10^−3^	2.0 × 10^−3^	0.005
Rurality index	−9.6 × 10^−3^	1.3 × 10^−3^	<0.001
Intake of polyunsaturated fats (g/1000 kcal)	−6.8 × 10^−4^	2.1 × 10^−4^	0.001

Table 3 displays the results of the multivariate linear regression model with fasting CBG as the dependent variable. BMI, WHR, and tobacco consumption were positively associated with fasting CBG, while higher physical activity was associated with lower fasting CBG (Table 3). The untransformed model was heteroscedastic and lacked normality of standardized residuals. Using the Box Cox function in STATA and testing various transformations yielded no improvement. We therefore present the original untransformed model with robust standard errors [24]. The adjusted R-squared value for the model was 0.10.

**Table 3:** Factors associated with fasting capillary blood glucose for a sample of individuals from rural south India

Variable	Coefficient	Robust standard error	*p-*value
Constant	1.28	0.943	0.176
Physical activity score (h/day of moderate physical activity	−0.056	0.015	<0.001
Body mass index (standardized to one unit SD)	0.41	0.11	<0.001
Waist-to-hip ratio (standardized to one unit SD)	0.17	0.087	0.046
Tobacco consumption (Y/N)	0.42	0.16	0.011

The following variables were associated (*p*-value <0.05) with pre-diabetes (IFG and/or IGT) in univariate logistic regression analyses: standardized BMI [OR 1.62, 95 % CI 1.25, 2.10], standardized WHR [OR 1.55, 95 % CI 1.20, 2.01], physical activity [OR 0.90, 95 % CI 0.84, 0.98], rurality index [OR 0.67, 95 % CI 0.56, 0.79], sedentary time [OR 1.09, 95 % CI 1.01, 1.20], TV time [OR 1.21, 95 % CI 1.02, 1.45], hypertension [OR 1.88, 95 % CI 1.11, 3.19], livestock ownership [OR 0.55, 95 % CI 0.32, 0.94], family history of diabetes [OR 3.1, 95 % CI 1.53, 6.27], pucca house [OR 2.11, 95 % CI 1.11, 4.03], and fat intake [OR 0.92, 95 % CI 0.86, 0.99].

The following variables were associated (*p-*value <0.05) with newly-diagnosed diabetes in univariate logistic regression analyses: age [OR 1.02, 95 % CI 1.00, 1.04], standardized BMI [OR 2.18, 95 % CI 1.65, 2.89], standardized WHR [OR 2.02, 95 % CI 1.53, 2.65], physical activity [OR 0.78, 95 % CI 0.70, 0.87], rurality index [OR 0.71, 95 % CI 0.60, 0.85], sedentary time [OR 1.20, 95 % CI 1.09, 1.32], tobacco consumption [OR 2.08, 95 % CI 1.16, 3.71], in-house tap water [OR 2.5, 95 % CI 1.10, 5.71], hypertension [OR 2.8, 95 % CI 1.55, 4.95], livestock ownership [OR 0.45, 95 % CI 0.24, 0.86], and Muslim religion [OR 3.2, 95 % CI 1.03, 10.11].

After multivariate adjustment, several risk factors were associated with odds of pre-diabetes and newly-diagnosed diabetes (Table 4). Physical activity was associated with lower odds of pre-diabetes and diabetes. Family history of diabetes was associated with increased odds of pre-diabetes but not diabetes. BMI and WHR were both independently associated with increased odds of diabetes. Higher rurality indices were associated with lesser odds of both pre-diabetes and diabetes. Tobacco consumption was associated with increased odds of diabetes. Intake of polyunsaturated fatty acids (PUFA) was associated with decreased odds of pre-diabetes and diabetes. PUFA consumption also confounded the association between family history and both pre-diabetes and diabetes, based on a change in coefficient of greater than 20 % after its removal from the model. This confounding effect suggests that there may be an interaction with family history, possibly due to household clustering of dietary factors.

**Table 4:** Factors associated with pre-diabetes and type 2 diabetes among a sample of individuals in rural south India, normoglycemia as referent

Variable	Pre-diabetes (IFG and/or IGT) OR and 95 % CI	Type 2 diabetes OR and 95 % CI
Physical activity (h/day of moderate physical activity)	0.96 (0.88, 1.04)	0.81^a^ (0.72, 0.91)
Family history of diabetes (Y/N)	3.08^a^ (1.21, 6.71)	1.74 (0.65, 4.66)
Body mass index (standardized to one unit SD)	1.28^d^ (0.94, 1.75)	1.85^a^ (1.33, 2.56)
Waist to hip ratio (standardized to one unit SD)	1.33^c^ (0.99, 1.80)	1.62^a^ (1.18, 2.56)
Rurality index	0.60^a^ (0.48, 0.75)	0.76^b^ (0.60, 0.97)
Total polyunsaturated fat consumption (g/1000 kcal)	0.92^a^ (0.88, 0.97)	0.94^b^ (0.90, 0.99)
Tobacco consumption (Y/N)	1.52^d^ (0.85, 2.74)	2.82^a^ (1.47, 5.41)

^a^ Significant to *p* < 0.01

^b^ Significant to *p* < 0.05

^c^ Tendency to *p* < 0.1

^d^ Tendency to *p* < 0.2

## Discussion and conclusion

The age- and sex-standardized prevalence of diabetes in the research site was 10.8 %, which is one of the highest recorded prevalence rates in rural India [3]. Vijayakumar et al. (2009) found higher prevalence of diabetes in a rural region of neighbouring Kerala based on fasting plasma glucose [25]. Chow et al. (2006) also found a higher prevalence in a rural area of neighbouring Andhra Pradesh, however the sample population comprised only of individuals >30 years of age [26]. Similar prevalence rates have been found in peri-urban villages in Tamil Nadu [27]. All recent cross-sectional studies in rural Tamil Nadu found prevalence rates between 5.1 and 8.4 %, slightly lower than the current study [19, 28, 29]. High prevalence in our sample may be indicative of local disparities and/or a continued increase of rural diabetes as predicted by Misra et al. [3]. Our study adds to the growing body of evidence suggesting that diabetes is no longer confined to urban areas of India and is a serious concern in rural regions as well. This is particularly troubling considering over 70 % of the population of India lives in rural regions and these areas are often characterized by widespread poverty and poor access to health care services [30].

Over half (56.4 %) of individuals with diabetes were previously undiagnosed, of which only 10 % were aware of the disease. The ratio of known to newly diagnosed diabetes was similar to a recent study in rural Tamil Nadu conducted by Anjana et al. (2011) (48 % undiagnosed) [19], but markedly lower than a different large-scale cross-sectional study by Misra et al. (2011) (25 % undiagnosed) [32], indicating a low level of diabetes awareness among the study population and discrepancies in the coverage of screening programs.

Age- and sex-standardized prevalence rates of impaired fasting glucose and impaired glucose tolerance were 3.9 and 5.6 % respectively, which sum to a pre-diabetes prevalence of 9.5 %, similar to other recent studies in rural Tamil Nadu [19, 28, 31]. Balagopal et al. (2008) found a markedly higher prevalence of pre-diabetes (13.5 % based on fasting glucose only), indicating regional disparities [29]. 50 % of individuals with pre-diabetes will develop overt diabetes within 10 years [32]. Consequently, high rates of pre-diabetes expose the potential for a continued rise of diabetes in the coming years, which may be of particular concern for already overburdened health systems in rural areas.

Previous studies have reported on various causes of diabetes in India, including urbanization, the ‘nutrition transition’, decreased physical activity, and emotional stress [33]. The changing dietary profile of Indian populations has received attention recently, including, most notably, increased consumption of ‘westernized’ foods and declining popularity of traditional coarse cereals [34, 35]. However, to our knowledge, this is the first cross-sectional study to assess associations between a wide range of dietary and lifestyle risk factors and diabetes outcomes among new diagnoses in a rural region. While other studies have attempted to correlate risk factors with diabetes outcomes, most have been in an urban environment [36, 37], only included self-reported diabetes [38], or failed to account for multiple variables and confounders in a multivariate statistical model [39–41]. This research is therefore important to elucidate the unique epidemiological environment of rural areas and identify the distinct factors associated with rural diabetes.

We found that higher physical activity was associated with lower post-glucose-load CBG, lower fasting CBG, and lesser odds of diabetes [OR 0.79]. This finding is consistent with previous research on the protective effects of physical activity against obesity, cardiovascular disease, and the metabolic syndrome [42]. The associations remained even after controlling for anthropometric measures, indicating that physical activity may have a direct impact on risk of diabetes apart from its association through overweight or obesity [43].

Higher BMI and WHR were independently associated with higher post-glucose-load CBG, fasting CBG, and greater odds of diabetes. While overweight and obesity have been substantiated as risk factors for cardiovascular disease and the metabolic syndrome [44, 45], ours is the first cross-sectional study in rural India to identify significant associations of BMI and WHR independent of each other. Currently, there is debate as to whether BMI or WHR ratio is better at conferring risk of non-communicable diseases [46]. Some recent studies have found that the association between BMI and diabetes becomes non-significant when adjusting for WHR and other variables, indicating that WHR is a better predictor of risk for Asian populations [47–49]. By contrast, a meta-analysis by Vazquez et al. (2005) found no significant difference in the abilities of BMI and WHR to predict incident diabetes, however they did find regional differences in pooled risk ratios, indicating disparity due to ethnicity [46]. In our analysis, both BMI and WHR were significant in all multivariate models and coefficients and ORs did not differ significantly. We therefore suggest that general obesity (i.e. BMI) and central obesity (i.e. WHR) may both be important risk factors for diabetes among Indian populations and should be considered independently and concurrently in research and clinical settings [50].

Tobacco (smoking and/or smokeless) consumption was prevalent among 48.7 % of men and 30.6 % of women. This corresponds with most previous studies that have found higher rates of use among men than women in India, but overall prevalence differs between regions [51]. Tobacco consumption was associated with higher fasting CBG and greater odds of diabetes. Smoking has long been associated with glucose intolerance and diabetes. A systematic review by Willi et al. (2007) found that among 25 observational studies, all but one identified a positive association between smoking and diabetes [52]. Cohort studies have also found a dose–response relationship between cigarettes/day and incidence of diabetes [53, 54]. Smokeless tobacco use among the study population is also concerning. A study in Sweden showed that smokeless tobacco users were three times more likely to develop diabetes than non-users [55]. In addition, most users in India mix smokeless tobacco with betel nut (*areca catechu*), which is independently associated with risk of diabetes [56]. With over one-third of the study population currently consuming tobacco, its contribution to chronic disease burdens cannot be underestimated. Reducing tobacco consumption must form an integral component of all prevention programs for diabetes, cardiovascular disease, cancers, and other non-communicable diseases [57].

Urban status is associated with diabetes risk in India [8, 28, 58]. Previous population-based chronic disease studies in India have maintained the dichotomous urban and rural definitions employed by the Census of India [59]. This dichotomous typology oversimplifies the urban/rural continuum and fails to capture the range of variation within rural areas [12]. While the entire study population was classified as ‘rural’ as per the Census of Indian definition, we aimed to examine the variability within the study region by employing a rurality index. Surprisingly, rurality was one of the strongest predictors of diabetes risk outcomes. Lower rurality was associated with higher post-glucose-load CBG and higher odds of pre-diabetes and diabetes. We therefore posit that the rurality index captured variability in socioeconomic, lifestyle, and dietary factors not fully represented by other variables. More specifically, we suggest that wealth, lifestyle, diet, and culture differs with proximity to the market village and size of home village, which in turn impacts an individual’s risk of diabetes. Moving forward, we recommend that researchers and policymakers examine the rural/urban interface more closely.

Another interesting finding was the association between intake of PUFAs and diabetes outcomes. PUFA intake appeared to confound the association between family history and both pre-diabetes and newly-diagnosed diabetes, likely due to clustering of dietary intake variables within households. We must therefore be careful in interpreting these variables in the regression models. Nonetheless, intake of PUFAs was associated with a lower fasting CBG and lesser odds of pre-diabetes and diabetes. This was the only dietary factor that had a significant association with any outcome in the multivariate models. PUFAs are primarily found in natural vegetable oils, whole grains, nuts, seeds, and fish. While more research is needed in this area, this finding corresponds with a review study done by Hu et al. (2001), which concluded that substituting unhydrogenated unsaturated fats for saturated fats and trans-fats could lower risk of diabetes and other chronic diseases [60]. Apart from this study, little research has examined the association between dietary fat intake and diabetes. Results suggest that further investigations that better control for household clustering of both genetic and dietary factors are necessary.

The lack of results showing significant associations between dietary variables and diabetes outcomes is notable, especially considering the overwhelming evidence of the importance of dietary risk factors in determining risk of obesity, diabetes, and the metabolic syndrome [3, 5, 8, 33, 35, 36, 38, 61]. This may expose limitations in the ability of the FFQ to adequately assess dietary factors due to recall bias, interviewer bias, or location-specific anomalies. However, it is more likely that results reflect a lack of sufficient variability in the rural diet to adequately assess the associations between dietary intakes and glucose tolerance within the study population. This is an important finding, as it may indicate that, while dietary changes are driving the diabetes epidemic in other locations, such as urban India, the rising rural prevalence of diabetes is due to other risk factors, such as physical activity, rising obesity, and genetic predisposition. Alternatively, individual dietary intakes may be changing uniformly among populations in rural India, perhaps due to effects of the nutrition transition, thus increasing risk of pre-diabetes and diabetes across the population as a whole [36, 37]. Thus, future observational studies examining dietary factors should ensure that the study population is heterogenous in its dietary intake, and should take into account the shifting nature of diet and nutrition in rural south India.

Our study has a number of limitations. Most importantly, the cross-sectional nature of the study precludes the ability to distinguish causes from effects. However, we hope that exclusion of individuals with pre-diagnosed diabetes from the models reduced reverse-causation. Due to limited access to laboratories and transportation constraints, we used CBG instead of venous plasma BG, which has a wider coefficient of variation [19]. However, there is good correlation between CBG and venous plasma estimations [16]. In addition, there has been an increasing emphasis on the associations between diabetes and stress, including co-morbid depression [62, 63]. Unfortunately, due to staffing and time constraints, we were unable to assess emotional stress or depression during the questionnaire, and therefore no measure of stress was included in the analyses. This is a notable exception, as stress may be a modifier or confounder for other putative risk factors such as socioeconomic position, obesity, rurality, and diet. Finally, our study sample comprised a small population of adults living in a rural region of Tamil Nadu. It is not nationally representative, and thus findings are not generalizable to the entire population of India.

Research and public health implications of the present study are considerable. Despite 70 % of India’s population living in rural areas, this portion of the population has largely been ignored by diabetes researchers, who focus their efforts on the urban ‘hot-spots’, where research is logistically easier and prevalence rates are higher. Our study contributes to the growing body of research suggesting that diabetes is rapidly growing in rural areas and must be addressed. In addition, we have identified a number of potential risk factors, including physical activity, family history, central obesity, abdominal obesity, rurality, polyunsaturated fat consumption, and tobacco consumption, which are all associated with diabetes indicators, pre-diabetes, and/or diabetes in the research site and warrant future investigation. Research such as this contributes to decision-making regarding the allocation of scarce public health resources and public health education programs to reduce diabetes burdens in rural regions of India.

## Appendix 1: List of putative risk factors investigated in regression models

*Non*-*dietary*

Age, [age]^2^, sex, rurality index, wealth index, family history of diabetes, body mass index, waist-to-hip ratio, waist circumference, physical activity score, television screen time, sedentary time, (physical activity)*(television screen time), (physical activity)*(sedentary time), grade achieved, participation in NREGA, local business owner, day labourer, high caste, low caste, vehicle ownership, television ownership, land ownership, [land ownership]^2^, migrant labourer, water source, hypertension, livestock owner, Hindu, Muslim, Christian, type of house (kutcha, semi-pucca, or pucca), smoking, smokeless tobacco user, general tobacco user (smoking or smokeless combined), total energy intake.

*Dietary*—*intake of items (g/1000* *kcal consumed)*

Carbohydrates, proteins, fats, millets, refined grains, whole wheat, leafy vegetables, tuber vegetables, meat, poultry, nuts and seeds, pulses, fruits, milk products, refined sugar, salt, total dietary fibre, insoluble dietary fibre, soluble dietary fibre, total fats, total oils, saturated fatty acids, polyunsaturated fatty acids, monounsaturated fatty acids, alcohol,

## Abbreviations

BMI: body mass index
CBG: capillary blood glucose
FFQ: food frequency questionnaire
GPAQ: Global Physical Activity Questionnaire
IFG: impaired fasting glucose
IGT: impaired glucose tolerance
LMIC: low- and middle-income country
MDRF: Madras Diabetes Research Foundation
OR: odds ratio
PUFA: polyunsaturated fatty acid
SES: socioeconomic status
WHO: World Health Organization
WHR: waist-to-hip ratio

## References

[1] International Diabetes Federation. IDF Diabetes Atlas, 6th ed. 2014. http://www.idf.org/sites/default/files/EN_6E_Atlas_Full_0.pdf. Accessed 23 May 2015.

[2] Hwang CK, Han PV, Zabetian A, Ali MK (2012). Venkat Narayan KM. Rural diabetes prevalence quintuples over twenty-five years in low- and middle-income countries: a systematic review and meta-analysis. Diabetes Res Clin Pract.

[3] Misra P, Upadhyay RP, Misra A, Anand K (2011). A review of the epidemiology of diabetes in rural India. Diabetes Res Clin Pract.

[4] Nichols GA, Hillier TA, Brown JB (2007). Progression from newly acquired Impaired Fasting Glucose to Type 2 diabetes mellitus. Diabet Care.

[5] Ajay V, Prabhakaran D, Jeemon P, Thankappan K, Mohan V, Ramakrishnan L (2008). Prevalence and determinants of diabetes mellitus in the Indian industrial population. Diabet Med.

[6] World Health Organization. WHO STEPS Surveillance Manual: The WHO STEPwise approach to chronic disease risk factor surveillance. 2005. http://apps.who.int/iris/bitstream/10665/43376/1/9241593830_eng.pdf?ua=1. Accessed 10 Dec 2014.

[7] International Institute for Population Sciences (IIPS). National Family Health Survey (NFHS-2). Mumbai: IIPS; 2007.

[8] Ebrahim S, Kinra S, Bowen L, Andersen E, Ben-Shlomo Y, Lyngdoh T, Ramakrishnan L (2007). The effect of rural-to-urban migration on obesity and diabetes in India: a cross-sectional study. PLoS Med.

[9] Sudha V, Radhika G, Sathya RM, Ganesan A, Mohan V (2006). Reproducibility and validity of an interviewer-administered semi-quantitative questionnaire to assess dietary intake of urban adults in southern India. Int J Food Sci Nutr.

[10] Madras Diabetes Research Foundation. EpiNu^®^ version 1.0. Chennai; 2007.

[11] World Health Organization. Global Physical Activity Questionnaire (GPAQ) Analysis Guide. 2011. http://www.who.int/chp/steps/resources/GPAQ_Analysis_Guide.pdf. Accessed 10 November 2013.

[12] Humphreys JS (1998). Delimiting ‘rural’: implications of an agreed ‘rurality’ index for healthcare planning and resource allocation. Aust J Rural Health.

[13] Weinert C, Boik R (1995). MSU Rurality Index: development and evaluation. Res Nurs Health.

[14] Anjana MR, Pradeepa R, Deepa M, Datta M, Sudha V, Unnikrishnan R (2011). The Indian Council of Medical Research-India Diabetes (ICMR-INDIAB) Study: methodological Details. J Diabetes Sci Technol.

[15] World Health Organization. Definition and diagnosis of diabetes mellitus and intermediate hyperglycemia. 2006. http://www.who.int/diabetes/publications/Definition%20and%20diagnosis%20of%20diabetes_new.pdf. Accessed 29 Nov 2013.

[16] Priya M, Anjana RM, Pradeepa R, Jayashri R, Deepa M, Bhansali A, Mohan V (2011). Comparison of capillary whole blood versus venous plasma glucose estimations in screening for diabetes mellitus in epidemiological studies in developing countries. Diabetes Technol Ther.

[17] Bhavadharini B, Mahalakshmi MM, Maheswari K, Kalaiyasari G, Anjana RM, Deepa M (2015). Use of capillary blood glucose for screening for gestational diabetes mellitus in resource-constrained settings. Acta Diabetol.

[18] Rose GA, Blackburn H, Gillum RF, Prineas RJ. Cardiovascular Survey Methods, 2nd ed. WHO Monograph Series No 56. 1982; Geneva: World Health Organization. 4972212

[19] Anjana RM, Pradeepa R, Deepa M, Datta M, Sudha V, Unnikrishnan R (2011). Prevalence of diabetes and prediabetes (impaired fasting glucose and/or impaired glucose tolerance in urban and rural India: phase I results of the Indian Council of Medical Research—INdia DIABetes (ICMR-INDIAB) study. Diabetologia.

[20] World Health Organization. The Asia Pacific perspective. Redefining obesity and its treatment. World Health Organization. International Association for the study of Obesity and International Obesity Task Force. Melbourne International Diabetes Institute; 2000.

[21] StataCorp: College Station TX 1985–2013.

[22] Census of India 2011 database. Government of India, Ministry of Home Affairs, Office of the Registrar General and Census Commissioner. 2011. http://www.censusindia.gov.in/2011census/C-series/C-13.html. Accessed 15 Jan 2015.

[23] Dohoo I, Martin W, Stryhn H, Dohoo I, Martin W, Stryhn W (2009). Linear regression. Veterinary Epidemiologic Research.

[24] Pires AM, Rodrigues IM (2007). Multiple linear regression with some correlated errors: classical and robust methods. Stat Med.

[25] Vijayakumar G, Arun T, Kutty VR (2009). High prevalence of type 2 diabetes mellitus and other metabolic disorders in rural central Kerala. J Assoc Physicians of India.

[26] Chow C, Cardona M, Raju PK, Iyengar S, Sukumar A, Raju R (2007). Cardiovascular disease and risk factors among 345 adults in rural India—the Andhra Pradesh Rural Health Initiative. Int J Cardiol.

[27] Ramachandran A, Mary S, Yamuna A, Nurugesan N, Snehalatha C (2008). High prevalence of diabetes and cardiovascular risk factors associated with urbanization in India. Diabet Care.

[28] Misra R, Misra A, Kamalamma N, Vikram NK, Gupta S, Sharma S (2011). Difference in prevalence of diabetes, obesity, metabolic syndrome, and associated cardiovascular risk factors in a rural area of Tamil Nadu and an urban area of New Delhi. Int J Diabetes Dev Ctries.

[29] Balagopal P, Kamalamma N, Patel TG, Misra R (2008). A community based diabetes prevention and management education program in a rural village in India. Diabet Care.

[30] Government of India. SRS Statistical Report. 2011. http://www.censusindia.gov.in/vital_statistics/SRS_Reports.html. Accessed 20 Feb 2015.

[31] Ramachandran A, Snehalatha C, Baskar ADS, Mary S, Sathish Kumar CK, Selvam S (2004). Temporal changes in prevalence of diabetes and impaired glucose tolerance associated with lifestyle transition occurring in the rural population in India. Diabetologia.

[32] Nichols GA, Hillier TA, Brown JB (2007). Progression from newly acquired Impaired Fasting Glucose to Type2 diabetes mellitus. Diabet Care.

[33] Mohan V (2004). Why are Indians more prone to diabetes?. J Assoc Physicians India.

[34] Shetty P (2002). Nutrition transition in India. Public Health Nutr.

[35] Kumar P, Dey MM (2007). Long-term changes in Indian food basket and nutrition. Econ Polit Weekly.

[36] Radhika G, Sathya RM, Ganesan A, Saroja R, Vijayalakshmi P, Sudha V, Mohan V (2011). Dietary profile of urban adult population in South India in the context of chronic disease epidemiology (CURES-68). Public Health Nutr.

[37] Ajay V, Prahakaran D, Jeemon P, Thankappan K, Mohan V, Ramakrishnan L (2008). Prevalence and determinants of diabetes mellitus in the Indian industrial population. Diabet Med.

[38] Agrawal S, Ebrahim S (2011). Prevalence and risk factors for self-reported diabetes among adult men and women in India: findings from a national cross-sectional survey. Public Health Nutr.

[39] Agrawal RP, Singh G, Nayak KC, Kochar DK, Sharma RC, Beniwal R (2004). Prevalence of diabetes in camel-milk consuming ‘Raica’ rural community of northwest Rajasthan. Int J Diab Dev Countries.

[40] Kokiwar PR, Gupta S, Durge PM (2007). Prevalence of diabetes in a rural area of central India. Int J Diab Dev Countries.

[41] Pandey RM, Gupta R, Misra A, Misra P, Singh V, Agrawal A (2013). Determinants of urban-rural differences in cardiovascular risk factors in middle-aged women in India: a cross-sectional study. Int J Cardiol.

[42] Kesaniemi YA, Danforth AE, Jensen MD, Kopelman PG, Lefebvre P, Reeder BA (2001). Dose-response issues concerning physical activity and health: an evidence-based symposium. Med Sci Sport Exec.

[43] Little M, Humphries S, Patel K, Dewey C. Factors associated with BMI, underweight, overweight, and obesity among adults in a population of rural south India: A cross-sectional study. 2015. BMC Obes. Manuscript submitted for publication (copy on file with author). 10.1186/s40608-016-0091-7 PMC4761187 26904203

[44] DeFronzo RA (2004). Pathogenesis of type 2 diabetes mellitus. Med Clin North Am.

[45] Mantzoros CS. Obesity and Diabetes Humana Press: Totowa.

[46] Vazquez G, Duval S, Jacobs DR, Silventoinen K (2007). Comparison of body mass index, waist circumference and waist/hip ratio in predicting incident diabetes: a meta-analysis. Epidemiol Rev.

[47] Cheng CH, Ho CC, Yang CF, Huang YC, Lai CH, Liaw YP (2010). Waist-to-hip ratio is a better anthropometric index than body mass index for predicting the risk of type 2 diabetes in Taiwanese population. Nutr Res.

[48] Kaye S, Folsom A, Sprafka J, Prineas R, Wallace R (1991). Increased incidence of diabetes mellitus in relation to abdominal obesity in older women. J Clin Epidemiol.

[49] Huxley R, James WP, Barzi F, Patel JV, Lear SA, Suriyawongpaisal P (2008). Obesity in Asia Collaboration. Ethnic comparisons of the cross-sectional relationships between measures of body size with diabetes and hypertension. Obes Rev.

[50] Stevens J, Couper D, Pankow J, Folsom AR, Duncan BB, Nieto FJ (2001). Sensitivity and specificity of anthropometrics for the prediction of diabetes in a biracial cohort. Obes Res.

[51] Kaur P, Rao SR, Radhakrishnan E, Ramachandran R, Venkatachalam R, Gupte MD (2011). High prevalence of tobacco use, alcohol use, and oberweight in a rural population in Tamil Nadu, India. J Postgrad Med.

[52] Willi C, Bodenmann P, Ghali WA, Faris PD, Cornuz J (2007). Active smoking and the risk of type 2 diabetes: a systematic review and meta-analysis. J Am Med Ass.

[53] Will JC, Galuska DA, Ford ES, Mokdad A, Calle EE (2001). Cigarette smoking and diabetes mellitus: evidence of a positive association from a large prospective cohort study. Int J Epidemiol.

[54] Rimm EB, Manson JE, Stampfer MJ, Colditz GA, Willett WC, Rosner B, Hennekens CH (1993). Cigarette smoking and the risk of diabetes in women. Am J Public Health.

[55] Persson PG, Carlsson S, Svanstrom L, Ostenson CG, Efendic S, Grill V (2000). Cigarette smoking, oral moist snuff use and glucose intolerance. J Int Med.

[56] Tung TH, Chiu RH, Chen LS, Wu HM, Boucher BJ (2004). Chen THH. A population-based study of the association between areca nut chewing and type 2 diabetes mellitus (Keelung Community-based Integrated Screening programme No. 2). Diabetologia.

[57] WHO. Report on the global tobacco epidemic. 2013. http://apps.who.int/iris/bitstream/10665/85380/1/9789241505871_eng.pdf?ua=1. Accessed 29 July 2015.

[58] Samuel P, Antonisamy B, Raghupathy P, Richard J, Fall CHD (2012). Socio-economic status and cardiovascuolar risk factors in rural and urban areas of Vellore, Tamilnadu, South India. Int J Epidemiology.

[59] Government of India. Census Terms. 2011. http://censusindia.gov.in/Data_Products/Library/Indian_perceptive_link/Census_Terms_link/censusterms.html. Accessed 31 March 2015.

[60] Hu FB, van Dam RM, Liu S (2001). Diet and risk of type II diabetes: the role of types of fat and carbohydrate. Diabetologia.

[61] Radhika G, Van Dam RB, Sudha V, Ganesan A, Mohan V (2009). Refined grain consumption and the metabolic syndrome in urban Asian Indians (Chennai Urban Rural Epidemiology Study 57). Metabolism.

[62] Anderson RJ, Freedland KE, Clouse RE, Lustman RJ. The prevalence of comorbid depression in adults with diabetes: A meta-analysis. Diabetes Care. 2001;24(6):1069-78. 10.2337/diacare.24.6.1069 11375373

[63] Roy T, Lloyd CE. Epidemiology of depression and diabetes: A systematic review. J Affect Disord. 2012;142(Suppl):S8-S21. 10.1016/S0165-0327(12)70004-6 23062861

